# When birth is not as expected: a systematic review of the impact of a mismatch between expectations and experiences

**DOI:** 10.1186/s12884-021-03898-z

**Published:** 2021-07-02

**Authors:** Rebecca Webb, Susan Ayers, Annick Bogaerts, Ljiljana Jeličić, Paulina Pawlicka, Sarah Van Haeken, Nazihah Uddin, Rita Borg Xuereb, Natalija Kolesnikova, COST action CA18211:DEVoTION team, Susan Ayers, Annick Bogaerts, Rita Borg Xuereb, Ljiljana Jeličić, Paulina Pawlicka, Isabel Soares, Mirjana Sovilj, Stef Savona Ventura

**Affiliations:** 1 Centre for Maternal and Child Health Research, City, University of London, London, EC1V 0HB UK; 2 KU Leuven, Department of Development and Regeneration, Research Unit Women and Child, B-3000 Leuven, Belgium; 3 Faculty of Medicine and Health Sciences, Centre for Research and Innovation Care (CRIC), University of Antwerp, Antwerp, Belgium; 4 Faculty of Health, University of Plymouth, Plymouth, Devon PL4 8AA UK; 5 Cognitive Neuroscience Department, Institute for Research and Development “Life Activities Advancement Center”, Belgrade, Serbia; 6 Department of Speech, Language and Hearing Sciences, Institute for Experimental Phonetics and Speech Pathology, Belgrade, Serbia; 7 Department of Social Sciences, Institute of Psychology, University of Gdańsk, Gdańsk, Poland; 8 Research and Expertise, Resilient People, University College Leuven-Limburg, Diepenbeek, Belgium; 9 Department of Midwifery, Faculty of Health Sciences, University of Malta, Msida, Malta

**Keywords:** Birth expectations, Birth experiences, Birth plans, Mismatch, Psychological outcomes, Birth satisfaction

## Abstract

**Background:**

Pregnancy and childbirth are significant events in women’s lives and most women have expectations or plans for how they hope their labour and birth will go. It is possible that strong expectations about labour and birth lead to dissatisfaction or other negative outcomes if these expectations are not met, but it is not clear if this is the case. The aim was therefore to synthesise prospective studies in order to understand whether unmet birth expectations are associated with adverse outcomes for women, their partners and their infants.

**Method:**

Searches were carried out in Academic Search Complete; CINAHL; Medline; PsycINFO, PsychArticles, PubMed, SCOPUS and Web of Science. Forward and backward searches were also completed. Studies were included if they reported prospective empirical research that examined the association between a mismatch in birth expectations/experience and postnatal outcomes in women, their children and/or their partners. Data were synthesised qualitatively using a narrative approach where study characteristics, context and methodological quality were extracted and summarised and then the differences and similarities among studies were used to draw conclusions.

**Results:**

Eleven quantitative studies were identified for inclusion from nine countries. A mismatch between birth expectations and experiences was associated with reduced birth satisfaction. Three studies found a link between a mismatch and the development of postnatal post-traumatic stress disorder (PTSD). The evidence was inconsistent for postnatal depression, and fear of childbirth. Only one study looked at physical outcomes in the form of health-related quality of life.

**Conclusions:**

A mismatch between birth expectations and experiences is associated with birth satisfaction and it may increase the risk of developing postnatal PTSD. However, it is not clear whether a mismatch is associated with other postnatal mental health conditions. Further prospective research is needed to examine gaps in knowledge and provide standardised methods of measuring childbirth expectations-experiences mismatch. To ensure women’s expectations are met, and therefore experience a satisfying birth experience, maternity providers should provide sensitive care, which acknowledges women’s needs and preferences, is based on open and clear communication, is delivered as early in pregnancy as possible, and enables women to make their own decisions about care.

**Trial registration:**

**Protocol registration:** PROSPERO CRD42020191081.

**Supplementary Information:**

The online version contains supplementary material available at 10.1186/s12884-021-03898-z.

## The impact of a mismatch between expectations and experiences of birth on postnatal outcomes: A systematic review.

Pregnancy and childbirth are significant events in women’s lives that can be associated with both positive and negative emotions. Research suggests that childbirth may affect a woman’s sense of self [1, 2], and her physical and psychological wellbeing [3, 4]. A positive birth experience can provide women with feelings of satisfaction and empowerment [5, 6], whereas a negative birth experience can lead to feelings of disappointment [7], the delay of subsequent pregnancies [8], and in some cases the development of postnatal psychological difficulties such as post-traumatic stress disorder (PTSD) [9, 10] or depression [11], which are in turn associated with poor offspring developmental and psychological outcomes [12–23].

Pregnant women may form beliefs about what to expect during labour and birth. These expectations may be developed from antenatal education, books, television, the internet, healthcare providers or family and friends [24–29]. Many expectations about birth may also be in line with the belief system a woman holds about birth. One model, (the technocratic model) views childbirth as inherently risky, with the foetus being separate from the mother, the health of the infant during pregnancy and labour needing to be ensured through tests, and medication, and birth needing to be completed within a “safe” time window [30]. On the other hand, the holistic model of birth sees the mother and foetus as one, labour being a normal physiological process which generally does not require intervention and that happens at its own rate [30]. The development of these belief systems are likely to come from a range of sources, including previous birth experiences [31], and are often associated with the type of birth a woman may choose [32, 33].

Based on their expectations and beliefs, some women create specific birth plans, which may include preferences for how each stage of labour should be managed, pain relief options and immediate postnatal care, such as skin to skin contact [34]. However, the development and use of birth plans is a contentious issue for both women and healthcare professionals. For example, an analysis of online UK parenting forums found that some women believe that the idea of planning birth is problematic because labour and birth can be unpredictable [35]. Further, a qualitative study of nine maternity professionals found that midwives believe the term “birth plan” can be misleading, and may contribute to women having unrealistic expectations during labour [36]. Additionally, a qualitative study of women and healthcare professionals found that respondents believed having strong expectations and preferences during labour may lead to disappointment or dissatisfaction if these expectations are not met [37]. However, some of the respondents from these studies believed that clear birth plans or expectations can be used as a tool for communication between women and healthcare professionals and can therefore be beneficial [35, 37].

Cross-sectional research supports the idea that unmet birth expectations can affect birth satisfaction. For example, a survey of 442 postnatal women found that those whose birth plans were followed had significantly higher birth satisfaction levels [38]. On the other hand, a survey of over 2500 women found that those who had unexpected medical complications during birth were more likely to rate their childbirth experience as negative [39]. Qualitative data also supports these findings. For example, one study found that for women to perceive their birth as positive, they had to achieve their birth expectations [40]. Similarly, qualitative data from 115 women identified that where care had gone beyond expectations, birth satisfaction was increased [41]. However, as birth expectations in these studies were measured after women had given birth the results may be subject to recall bias and may not give an accurate representation of women’s expectations prior to birth, making conclusions difficult to draw.

The summarised research gives an unclear picture about whether having clear birth expectations can be beneficial to women’s birth experiences in terms of improved communication [35, 37], or lead to dissatisfaction or disappointment if expectations are not met [38–41]. Furthermore, many of the studies are cross-sectional surveys, where birth expectations are measured after women have given birth. These studies may therefore be subject to recall bias, and results may differ if women are asked about their birth expectations prior to giving birth and their experience after birth, so a prospective measure of any mismatch could be calculated.

Given the adverse impact a negative birth experience can have on women and their infants’ [7, 9–11, 19–21], it is important for us to understand whether having unmet birth expectations can contribute to negative birth experiences, and in turn, if these impact postnatal outcomes. Understanding the impact of unmet birth expectations on postnatal outcomes (such as satisfaction, mental health, physical wellbeing etc.) could help to identify problem areas that should be improved [42] and therefore lead to the improvement of maternity care for women and their partners. Therefore, the aims of this literature review are to: 1) review the evidence on the impact of a mismatch between birth expectations and experience on postnatal outcomes for the mother, her partner and her infant using prospective studies, and 2) provide a critical appraisal and overview of the evidence base.

## Method

### Protocol and registration

The protocol was registered with PROSPERO (CRD42020191081).

### Information sources and search

Literature searches and study selection were reported according to the Preferred Reporting Items for Systematic Reviews and Meta-analyses (PRISMA) guidelines [43]. Online databases were used to identify papers. Boolean operators (AND/OR) were used to combine subject headings and relevant search terms (see Table 1). Searches were limited to key words being present in the title of papers. The date of the last search was 6th April 2020. Forward and backward searches of included studies were carried out and completed by the 25th June 2020.

**Table 1:** Inclusion criteria and search strategy

Inclusion criteria and example search terms		
Criteria	Specification	Search terms
Population	Perinatal women (pregnancy to 1 year postnatal)	pregnancy OR pregnant OR pre-nat* OR prepart* OR ante-nat* OR antenat* OR ante-part* OR peri-nat* OR perinat* OR peri-part* OR peripart* OR puerper* OR post-nat* OR postnat* OR post-part* OR postpart* OR mother* OR birth OR childbirth
Observation	A mismatch between birth expectations (measured in pregnancy) and birth experiences (measured after birth). The mismatch could refer to: procedures performed during birth, pain relief, feelings of control, emotions felt during birth. The mismatch must have been calculated through the use of discrepancy scores, or grouping of participants (i.e. labour/birth expectations were the same as labour/birth experiences (match) vs labour/birth expectations were different to labour/birth experiences (mismatch).	expect* OR belief OR desire OR predict* OR prefer*) AND (experienc* OR perception* OR perceiv* OR mismatch OR discrepancy OR satisfaction OR outcome OR mode OR “Obstetric outcome”)
Outcome	Postnatal outcomes in women, their partners and/or their children (aged 0–5 years). These can be psychological or physical.	health OR wellbeing OR problem* OR mental OR emotion* OR psychiatr* OR anxi* OR depress* OR affect* OR trauma OR PTSD OR stress OR ASD OR Disorder* OR illness OR symptom* OR breast* OR relationship OR bonding OR attachment OR child* OR infant
Study design	Empirical research. Intervention papers were included if they had baseline information (pre-intervention) or a ‘treatment as usual’ control group	
**Information sources**		
• EBSCO Host:		
■ Academic Search Complete (1887- present)		
■ CINAHL (1982- present)		
■ Medline (1946- present)		
■ PsychArticles (1967- present)		
■ PsycINFO (1806 – present)		
• SCOPUS (2004 - present)		
• PubMed (1910-present)		
• Web of Science (1970 – present):		
■ Science Citation Index Expanded (1970-present)		
■ Social Sciences Citation Index (1970-present)		
■ Art & Humanities Citation Index (1975-present)		
■ Conference Proceedings Citation Index-Science (1990-present)		
■ Conference Proceedings Citation Index- Social Science & Humanities (1990-present)		
■ Book Citation Index– Science (2005-present)		
■ Book Citation Index– Social Sciences & Humanities (2005-present)		
■ Emerging Sources Citation Index (2015-present)		

### Study selection

Search results were imported into Eppi-Reviewer 4, where duplicates were removed and results were screened by title and abstract by NU based on inclusion criteria (see Table 1). A proportion (10%) of the results were double screened by PP. Decisions to include or exclude were concordant between reviewers in 97.5% of cases. Once title and abstract screening was complete, full text screening was carried out by RW. A proportion (10%) were double screened by PP and decisions to include or exclude were concordant between reviewers in 100% of cases. Disagreements for both title and abstract and full text screening were discussed and resolved by NU, PP, SA and RW.

### Data collection process and data items

Data extraction was carried out using Eppi-Reviewer 4 which allows for line-by-line coding. A new ‘codeset’ labelled ‘Data Extraction’ was created and contained every item to be extracted from the data (e.g. year of publication, country of study). Each study was read in full, and relevant parts of the text highlighted (for example the country of the study) and applied to the relevant code.

Data were extracted for the following domains: Study Characteristics (country, setting, design, aim); Sample Characteristics (recruitment, size, age, ethnicity, employment, education, children, socioeconomic status, mental health problems, obstetric details, other demographic details); Data collection (method, ethical approval; measure of mismatch; measure of postnatal outcomes; measure of other variables); and Results (mismatch; postnatal outcomes; other variables).

### Quality appraisal of individual studies

Methodology sections of included texts were assessed for quality using Joanna Briggs Critical Appraisal Tools for Cohort studies [44]. Each point on the checklists can be coded into Yes/No/Unclear/Not applicable. Items which were coded as ‘Yes’ were assigned a score of 1, items coded as ‘No’ or ‘Unclear’ were assigned a score of 0. The higher the score, the higher the quality of the study. Methodological assessment of studies was done by two raters: RW and SVH. Coders assigned the same score to studies 44.4% of the time. Most disagreements were due to one study [34], where there were disagreements on 6 out of the 9 items. The corresponding author of this study was contacted in September 2020 for further clarification of the methodology; however, no response was received before submission of the systematic review. When this study was removed from agreement calculations, coders assigned the same score to study 90.3% of the time. All disagreements were discussed, with particular attention paid to Mei et al. (2016) [34] and were resolved by RW and SVH. The final appraisal is based on agreed answers. See supporting information for more details.

### Synthesis of results

Studies were synthesised narratively by RW using the technique described by Lucas et al., (2007) [45]. First, study characteristics, context and methodological quality were extracted and summarised. Next, the differences and similarities among studies were used to draw conclusions across the studies. Due to the small number of included studies, and the heterogeneity of these studies, there was not enough data to conduct a meta-analysis.

## Results

### Study characteristics

**Fig. 1:**
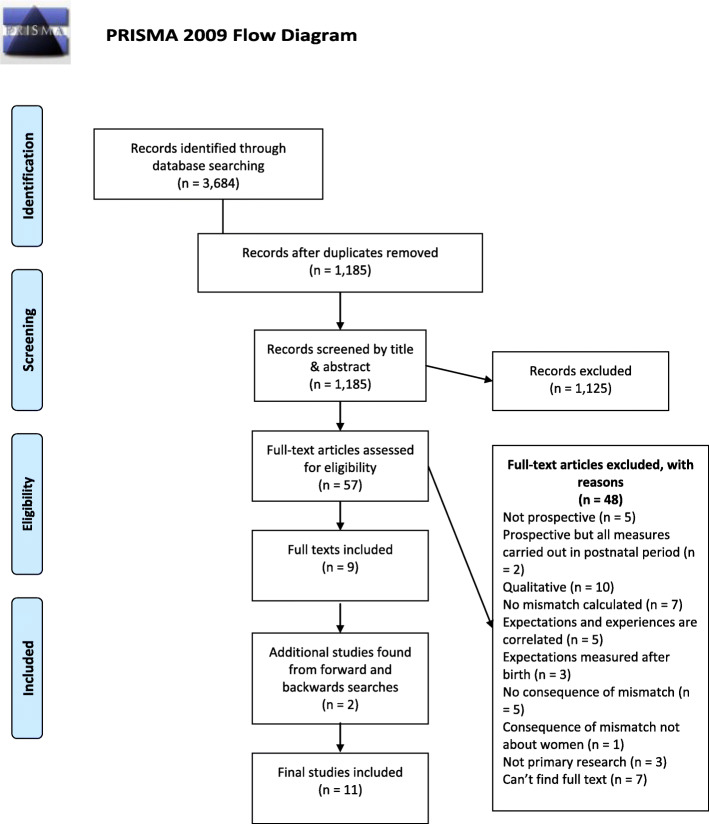
PRISMA Flow Diagram

Searches identified a total of 3684 citations. Hand searches of reference lists of included studies identified a further 2 studies. After removing duplicates and screening through abstracts, titles and full texts, 11 studies remained for inclusion in the review (Fig. 1). Studies included in the review are summarised in Tables 2 and 3. Half of the studies were carried out in English-speaking countries (Ireland *n* = 1; Canada *n* = 2, USA *n* = 4). One study [46] was carried out in multiple countries (Germany, Ireland and Italy). Sample sizes ranged from 30 to 1700 with an average of 401 participants. Six studies reported the average age of women as being between 25 and 32.21. Three studies reported that over half of women (63.8–83%) were aged between 26 and 35. Of the four studies that reported ethnicity, women were mainly white (47.5–95%) or Jewish (98.5%). The samples were well educated with most women having received 12+ years of education (54–96%). Nine of the studies were published within the last 10 years (Range: 1982–2020; Mean = 2011, Median = 2014).

**Table 2:** Study characteristics of included studies

#	Author & Year	Country of study	Study design	Sample size	Participant characteristics	Quality rating
	**Health related quality of life (*n*** **= 1)**					
1	Fobelets et al. (2019) [1]	Germany, Ireland, Italy	Prospective longitudinal (part of RCT called OPTIBIRTH [2])	862	All women had previous C-section; 83% aged 31 or over; 97% married or cohabiting; 38% birthing in Germany, 15% birthing in 47% birthing in Italy.	Good (Score: 7/9)
	**Psychological outcomes (*n*** **= 6)**					
2	Garthus-Niegel et al. (2014) [3]	Norway	Prospective cohort	1700	Age M = 31.2; 97.8% married or co-habiting; 47.2% first time mothers	Good (Score: 8/9)
3	Houston et al. (2015) [4]	USA	Prospective longitudinal	160	67.5% aged over 30; 47.5% White; 26.3% Black; 33.1% nulliparous; 30.6% previous c-section	Good (Score: 8/9)
4	Philipson-Price (1982) [5]	Canada	Cohort	30	Age M = 27.5	Medium (Score: 6/9)
5	Soet et al. (2003) [6]	USA	Prospective cohort	103	Mean age = 29.20 (SD = 4.99); 83% married; 67% white	Good (Score: 9/9)
6	Sluijs et al. (2020) [7]	The Netherlands	Prospective cohort	463	Most women (63.8–74%) aged between 26 and 35 years old; 98.2–98.3% of women married or cohabiting	Good (Score: 9/9)
7	Verreault et al. (2012) [8]	Canada	Prospective cohort	308	Age M = 32.21 (SD = 4.40); 97.1% married	Good (Score: 9/9)
	**Psychological outcomes and birth satisfaction (*n*** **= 2)**					
8	Stein De Luca & Lobel (2014) [9]	USA	Prospective longitudinal	164	Age M = 30; 95% White; 97% married or cohabiting; 23% previously given birth	Good (Score: 8/9)
9	Tanglakmankhong (2010) [10]	Thailand	Prospective longitudinal	195	Age M = 25; 51.8% first time pregnancy	Good (Score: 8/9)
	**Birth satisfaction (*n*** **= 2)**					
10	Mei et al. (2016) [11]	USA	Prospective cohort	94	NR	Poor (Score: 3/9)
11	Preis et al. (2019) [12]	Israel	Prospective longitudinal	330	Age M = 30, 98.5% Jewish, 95.2% married or cohabiting	Good (Score: 8/9)

**Table 3:** Measure of mismatch and results of included studies

#	Description of birth expectations	Time point	Description of birth experiences	Time point	Calculation of mismatch	Name & description of outcome measure	Time point	Findings
1	Women were asked: ‘right now, what type of birth would you like for this pregnancy?’ Answers were coded as follows: vaginal birth, caesarean birth, don’t know/unsure	Pregnancy	Actual mode of birth, from medical records	3 months after birth	Match VBAC-VBAC* (preference for VBAC, had VBAC) **Match ERCS-ERCS** (preference ERCS, had an ERCS), **Match ERCS-EMCS** (preference for elective caesarean section, had an Emergency caesarean), **Mismatch VBAC-ERCS** (wanted VBAC, had ERCS) **Mismatch VBAC-EMCS** (wanted VBAC, had EMCS) **No preference**	Health Related Quality of Life (HRQoL)- The Short Form-36 Health Survey version 2 (SF-36v2) [13]. This includes six dimensions: physical functioning, role limitation, social functioning, pain (bodily), mental health (psychological distress and psychological well-being) and vitality (energy/fatigue).	3 months after birth	Women with a match between their preference of birth and actual birth mode had higher HRQoL scores compared to women with a mismatch. Women with a “match VBAC-VBAC” had the highest HRQoL utility scores, and women in the “mismatch VBAC-ERCS” and “mismatch VBAC-EMCS” groups had the poorest.
2	Preference for a caesarean section (CS) based on the following question: “If I could choose, I would rather deliver by CS.” The answers were coded as: yes (“highly agree”, “agree”) or no (“disagree”, “highly disagree”).	32 weeks pregnant	Information on birth by elective CS was obtained from the hospital’s birth record.	After birth	**Match 1** (no preference for CS, no elective CS), **Match 2** (preference for CS, elective CS), **Mismatch 1** (no preference for CS, elective CS), and **Mismatch 2** (preference for CS, no elective CS).	Impact of events scale [14] which measures PTSD symptoms of intrusion and avoidance. Higher scores reflect a higher degree of post-traumatic stress, and a score above 34 on the Impact of Event Scale has been suggested to indicate that a PTSD condition is likely to be present.	8 weeks after birth	A significant interaction effect between preference and actual mode of birth (*F* = 7.15, *p* = .008) and PTSD symptoms. Bonferroni post-hoc tests found significant differences in PTSD symptoms between Match 1 (no preference for CS, no elective CS) and Mismatch 2 (preference for CS, no elective CS) Key findings from regression analysis were: (a) women who preferred CS, but whose actual mode of birth was vaginal, had a higher level of post-traumatic stress symptoms, and that (b) psychological factors such as fear of childbirth symptoms of depression, and anxiety were particularly potent risk factors that could explain parts of this effect.
3	Preferred birth – measured using a computerised standard gamble exercise where women were presented with a hypothetical choice of birth types and had to pick their preference for it (0 = least desired outcome; 1 = most preferred outcome).	24–36 weeks pregnant	Actual mode of birth	After birth	A score was calculated for women’s preference for vaginal birth (between 0 and 1). Preference for vaginal birth and actual mode of birth were used as predictors for postnatal depression symptoms.	Patient Health Questionnaire (PHQ-9) [15] which is used to assess depressive symptoms. Scores range from 0 to 27; higher scores indicate more depressive symptoms.	8–10 weeks and 6–8 months	There was a significant interaction between birth mode and vaginal birth preference on the PHQ-9 score at 8–10 weeks after birth (*p* = .047). Women with higher vaginal birth preference who had a CS birth had higher mean PHQ-9 scores at 8–10 weeks after birth (*p* = .027).
4	Labour and Delivery Scale [16] - Questions are answered on a 7-point scale (1 = negative – 7 = positive) regarding factors important to labour and birth.	5–9 months pregnant	Labour and Delivery Scale [16] Reworded to ask about how women experienced labour and birth.	2 days after birth	Scores were totalled and a discrepancy score was calculated by subtracting postnatal scores from prenatal score. Positive scores show that labour was more positive than expected, negative scores show labour was worse than expected	1. Multiple Affect Adjective Checklist (MAACL) [17] which measures positive and negative affect. Women were asked to mark “x” by the adjectives describing how they felt at the present moment. If more negative affect words were chosen women were assigned a negative score. 2. Distress rating scale (DRS [18]) was used to evaluate the discomfort caused by the physiological sensations experienced by women during birth.	2 days after birth	Positive discrepancy scores (i.e. women who experienced labour more positively than expected) were correlated with positive mood measured by MAACL (adjectives) and with low distress measured by DRS.
5	Wijma Delivery Expectance Questionnaire (WDEQ-A [19]) is a 33-item scale that measures women’s antenatal feelings and fears about childbirth.	Late pregnancy	Wijma Delivery Experience Questionnaire (WDEQ-B [19]) but is worded so that it can be completed after birth to assess fear of birth, feelings, and thoughts.	4 weeks after expected due date	To compute differences between a woman’s expectations and her actual experience, a difference score was calculated so that negative scores represented an experience that was more negative than expectations and positive scores revealed a more positive experience.	Traumatic Event Scale [20] was used to assess PTSD resulting from childbirth.	4 weeks after expected due date	Severity of pain in the first stage of labour, increased feelings of powerlessness, history of sexual trauma, **negative expectation difference**, less social support, increased medical intervention, lack of adequate information, higher expectations of pain, and length of labour were significant predictors of perceptions of the childbirth as traumatic. This model accounted for 55% of the variance in traumatic experience. T-test analyses also showed **expectation differences** were associated with perception of labour as traumatic.
6	Preferred mode of birth “If you could choose your mode of delivery, would you prefer a vaginal or Caesarean section”	30 weeks pregnant	Actual mode of birth Self-report and medical file data	8 weeks after birth	A new variable was constructed; the “Preference-Actual mode of delivery-Congruence” (PAC) variable resulting in four outcomes: (1) Preferred VD (vaginal delivery) - actual mode VD (2) Preferred VD- actual mode CS (3) Preferred CS - actual mode VD (4) Preferred CS - actual mode CS’	Fear of childbirth (FOC) WDEQ-A & WDEQ-B [19] was measured during pregnancy and after birth. The higher the sum score, the more severe is FOC. A sum score 85 indicates severe FOC, whereas a sum score 0–84 indicates none to moderate FOC.	30 weeks pregnant 8 weeks after birth	The results showed a significant interaction effect of time and PAC groups. The VD- > CS group showed less decrease of FOC scores from pre- to postnatal compared to other groups. Bonferroni post-hoc tests showed that the VD- > VD group, had the lowest mean FOC scores at both T1 and T2. The VD- > CS and CS- > VD groups had higher FOC scores at T2 scores than the VD- > VD group. When controlling for psychological variables (anxiety and depression scores during pregnancy) only the VD- > CS remained a significant predictor of higher FOC scores.
7	Wijma Delivery Expectancy Questionnaire (WDEQ-A) [19]	25–40 weeks pregnant	Wijma Delivery Experience Questionnaire (WDEQ-B) [19]	After birth (exact time point not clear)	A difference score was calculated between the two versions. Negative scores represented an experience that was more negative than expected and positive scores revealed a more positive experience than expected.	(a) The PTSD Module of Structured Clinical Interview [21] measured PTSD based on DSM-IV criteria. (b) The Modified PTSD Symptom Scale [22] which measured self-report PTSD symptoms based on DSM-III criteria.	3 & 6 months after birth	The women classified in the PTSD group reported a more negative childbirth experience than expected (*p* < .001). Other factors also associated with PTSD was less social support, higher trait anxiety, greater antenatal depression scores and a less positive perception of care received during labour
8	The degree of control women expected to have during labour and birth was measured by the 21-item Expectations About Childbirth Scale (EC1) designed by the authors of the paper. Participants rated their expectations of control during childbirth on a five-point Likert scale, with higher scores indicating greater expected control.	7–9 months pregnant	Experience of Childbirth Scale Questionnaire was the same measure but phrased in the past tense (e.g., “I had very little say over the way my labor and delivery went”) to assess perceived control during childbirth.	4–8 weeks after birth	The Unmet Expectations of Control variable was calculated by subtracting the scores from the postnatal questionnaire from the antenatal questionnaire.	(a) Childbirth Satisfaction Scale consisted of eight items such as “I am happy with my childbirth experience,” and “I wish my labor and delivery had gone differently than they did,” with a 5-point response scale (1 = Strongly Disagree, 5 = Strongly Agree) (b) Beck Depression Inventory [23] was used to measure depressive symptoms.	8 weeks after birth	Time waited to hold baby, perceived threat to self, perceived threat to baby, birth type, perceived control and **unmet expectations** of control all significantly predicted birth satisfaction. Prenatal depressed mood, childcare distress, support received from partner, concerns about the self and foetus during pregnancy and perceived control all predicted postnatal depressed mood. **Unmet expectations** of control did not predict postnatal depressed mood.
9	Thai Childbirth Expectations and Experiences Questionnaire (prenatal) Own scale, 36-items. Asked about the possible events that women think will happen during their labor and birth. The women were asked “do you think this situation will happen during your upcoming childbirth?” and answered “yes” or “no” to each question	3rd trimester of pregnancy	Thai Childbirth Expectations and Experiences Questionnaire (postnatal) women were asked to complete the second part of the questionnaire, which used the same set of items. Here the women were asked “did this situation happen during labor and birth?” and responded “yes” or “no”	2–3 days after birth	Each item was classified as: fulfilled expectations – women expected it to happen and it did happen unmet expectations – women expected it to happen, but it did not happen, unexpected experiences – women did not expect it to happen, but it did, and null experiences – women did not expect it to happen, and it did not happen.	(a) Satisfaction with childbirth experience (SCE) - women were asked for each of the 36 items: “how did you feel about what happened?” A 4-point response scale is used: 1 = not satisfied, 2 = low satisfied, 3 = moderately satisfied, 4 = very satisfied (b) Thai Childbirth Attitudes Questionnaire (TCAQ) was a 15-item scale adapted from a previous scale [24], that measured FOC.	2–3 days after birth	**Fulfilled expectations**, self-efficacy, and taking a childbirth class were significantly positively associated with SCE. **Fulfilled expectations** were a significant predictor of SCE (*p* < .001) in a regression analysis. Self-efficacy expectancy, childbirth attitudes and education were also significant predictors. FOC (measured after birth) was associated with a mismatch (i.e. when women did not expect the event to happen but it did) but this relationship was no longer significant after controlling for parity and complications during labour
10	Birth plan requests - Requests could be either a positive request (e.g. I would like delayed cord clamping) or a negative request (e.g. I do not want my baby to get eye ointment). Total number of specific requests was also recorded.	> 34 weeks pregnant	Medical record chart review	After birth	Matched plans to medical record.	Satisfaction with birth Women were asked to evaluate their hospital birth experience in three domains: 1) overall satisfaction with their birth experience, 2) if the birth experience was what they expected, and 3) if they felt in control of their birth experience. Questions were phrased as affirmative statements for each measure, and women agreed or disagreed with each statement on a Likert scale of 1–5.	After birth	Having a higher percentage of requests fulfilled significantly correlated with greater overall satisfaction (*p* = 0.03) and feeling that expectations were met (*p* < 0.01), Having a high number of requests (> 15) was associated with an 80% reduction in overall satisfaction with the birth experience compared with having 15 or fewer requests (*p* < 0.01)
11	Planned place and mode of birth- Women were asked how they planned to give birth (e.g. mode of birth, pain relief).	Pregnancy	Actual place and mode of birth- Women asked about what happened during their birth.	2 months after birth	Self-reports of the planned birth and actual birth were compared by calculating an incongruence score.	(a) Emotions during childbirth was measured using a scale designed by authors based on hotspots identified in Harris & Ayers (2012) [25] and WDEQ-B [19]. The presence of 27 different emotions during childbirth were used. (b) Global birth satisfaction was measured using the Childbirth Satisfaction Scale [26]. (c) Perceptions of care was measured using a tool developed for the study, based on the 3-item Patient Perception Score [27] and 10 additional items assessing interpersonal interactions with medical staff.	2 months after birth	Incongruence with birth plan was negatively associated with global birth satisfaction (*r* = −.13, *p <* .01). This relationship was also mediated by feelings of guilt (*r* = − 20, *p* < .001) during birth and perceptions of care (*r* = .17, *p* < .001).

### Risk of bias

Due to the lack of research in this area, an inclusive approach was taken, with no studies being excluded due to their quality. Nine out of the 11 studies were of good quality (scoring 7 out of 9 or more). One study was of medium quality (scoring 6) and one was low quality (scoring 3).

### Measurement of mismatch

Three studies [46–48] used women’s desired mode of birth and actual mode of birth to group women into matched vs mismatched groups (e.g. wanted a vaginal birth and had a vaginal birth = match vs wanted a vaginal birth and had a c-section = mismatch). One study [49] measured different aspects of labour and birth (e.g. medication, presence of family) and grouped women into four groups (1. fulfilled expectations - women expected an event to happen and it did happen; 2. unmet expectations – women expected an event to happen, but it did not happen; 3. unexpected experiences – women did not expect an event to happen but it did, and 4. null experiences – women did not expect an event to happen, and it did not happen). Another study [50] used a score to calculate women’s preference for a vaginal birth (0 = no preference, 1 = strong preference) and used this variable to assess the interaction with actual mode of birth on the outcome measure. One study [34] calculated the percentage of expectations fulfilled based on women’s own birth plans and their medical records. Two studies [51, 52] used the Wijma Delivery Expectancy Questionnaire (WDEQ-A [53]) to measure birth expectations and the Wijma Delivery Experiences Questionnaire (WDEQ-B [53]) to measure birth experiences. A difference score was calculated between the two where a negative score represented an experience that was more negative than expectations and positive scores revealed a more positive experience. The remaining studies [54, 55] calculated a discrepancy score based on what women wanted to happen during labour vs what actually happened or women’s expectations of control during labour and experiences of control during labour [56].

### Outcomes of mismatch

The outcomes of the mismatch were all measured for women. These were grouped into birth satisfaction (*n* = 2); psychological outcomes (*n* = 6); birth satisfaction and psychological outcomes (*n* = 2); and physical health (*n* = 1). No studies looked at the impact of a mismatch on women’s partners or baby/children.

### Birth satisfaction

Four studies looked at the impact of a mismatch between birth expectations and experiences on satisfaction with birth. Results suggest that if a woman had her birth expectations met, she was more likely to rate her birth experience as satisfying [34, 49]. Where birth expectations were not met childbirth satisfaction was low [55, 56]. As well as a direct relationship between a mismatch and childbirth satisfaction, one study [55] found an indirect relationship between a mismatch and childbirth satisfaction which was mediated by women’s perceptions of control, both negative and positive emotions and women’s perceptions of care. Furthermore, another study [49] showed that self-efficacy expectancy, childbirth class attendance and women’s education levels were also predictors of childbirth satisfaction. Additionally, one study found that birth plans with a lot of requests were associated with an 80% reduction in birth satisfaction [34].

### Psychological outcomes

Eight studies looked at the impact of a mismatch between birth expectations and experiences on psychological outcomes. Three studies looked at the impact of a mismatch on postnatal depression. Two studies found that a mismatch between birth expectations and experiences were not associated with postnatal depression symptoms [54, 56]. On the other hand, one study found an association between a mismatch in birth expectations/experiences and postnatal depressive symptoms [50]. Results indicated that in women who had a caesarean section (CS), higher preference for a vaginal birth was associated with higher postnatal depression scores. This suggests other factors are likely to be more important in postnatal depression than a mismatch between birth expectations and experiences. This is supported by another study [56] that showed prenatal depressed mood, childcare stress, satisfaction with social support received from their partner during birth, concerns about the self and the foetus during pregnancy, and perceived control during birth were associated with postnatal depressed mood whereas a mismatch was not.

Two studies looked at the impact of a birth expectations/experiences mismatch on fear of childbirth (FOC), again with mixed results. One study [49] found that FOC (measured after birth) was associated with a mismatch (women did not expect the event to happen but it did) but this relationship was no longer significant after controlling for parity and complications during labour. On the other hand, the other study [48] identified that women who wanted a vaginal birth but had a CS were at greater risk for severe FOC. This risk was not demonstrated for women who wanted a CS but had a vaginal birth.

Three studies looked at postnatal PTSD as an outcome of a mismatch between birth expectations and experiences. One study [47] showed that women who had a preference for a CS but did not receive one had significantly higher levels of PTSD compared to women who had no preference for a CS and did not receive one [47]. Furthermore, two studies [51, 52], found that more negative childbirth experiences than expected were associated with more PTSD symptoms after birth. However, both studies identified other factors that were also associated with the development of postnatal PTSD symptoms such as pain severity, powerlessness, history of sexual trauma, social support, medical interventions, length of labour [52], higher trait anxiety, greater antenatal depression scores and a less positive perception of care received during labour [51].

### Physical outcomes

One study [46] used a health-related quality of life (HRQoL) measure [57] to evaluate the impact of a mismatch between birth expectations and experiences on physical health in women who had previously had a CS. HRQoL measures subjective influence of the health status (physical and emotional) on daily functioning and not quality of life *per se* [58]. The authors found that women who had a match between their preferred mode of birth and actual mode of birth had higher HRQoL scores three months after birth. More specifically, women who wanted a vaginal birth after caesarean (VBAC) and had one, had the highest HRQoL scores. In contrast, women who wanted a VBAC but had an elective repeated CS or an emergency repeated CS had lower HRQoL scores.

## Discussion

This review aimed to understand the impact of a mismatch between labour/birth expectations and experiences on postnatal outcomes for the mother, her partner and her infant. The review identified 11 studies for inclusion. The majority of studies measured birth satisfaction or psychological symptoms as outcomes. Most studies were good quality, with only one rated as poor quality. Results from the review suggest that a mismatch between labour/birth expectations and experiences has a negative impact on women’s satisfaction with birth and may increase the risk of women developing PTSD after birth. There was not enough evidence to draw conclusions about postnatal depression, FOC or physical wellbeing.

The finding that a mismatch between birth expectations and experiences is associated with lower birth satisfaction is supported by similar research that was not included in this review, due to not meeting inclusion criteria. For example, several studies were excluded because they did not calculate a mismatch between birth expectations and birth experiences, but instead used a correlation (*n* = 2) [59, 60], did not calculate a mismatch (*n* = 2) [61, 62], or measured birth expectations and experiences at the same time [63, 64]. These studies also found that women who have a mismatch between birth expectations and experiences are less likely to rate their births as satisfying.

Results from the review also suggest that unmet expectations may increase women’s risk of developing PTSD after birth. However, all three studies examining this found that multiple other risk factors, such as fear of childbirth, depression, pain severity, feelings of powerless, lack of adequate information provision and previous trauma increased the chances of women developing PTSD [47, 51, 52]. This is in line with previous research that has demonstrated several factors can contribute to women’s risk of developing postnatal PTSD [39, 65–67]. This shows it is unlikely to be the unmet birth expectations themselves that lead to the development of postnatal PTSD, but a range of interrelated factors.

Results about the impact of unmet birth expectations on other psychological outcomes is less clear. Some studies suggested that unmet birth expectations are associated with psychological difficulties, such as depression [50], and FOC [48], whereas others found no relationship [49, 54, 56]. There are no clear patterns across the studies in terms of the number of women involved, the country of the study, the measure of the mismatch or the outcome measure used. This means it is not possible to hypothesise why some studies noted an association between unmet expectations and psychological outcomes and others did not. However, based on the results regarding birth satisfaction, it is possible that birth satisfaction might mediate the relationship between unmet expectations and psychological outcomes. It is also possible other factors may moderate the relationship (such as individual vulnerability e.g. previous trauma, interpersonal violence or obstetric risk).

### Implications for practice

The results from this review suggest that women should have their desires relating to labour and birth listened to in order to improve birth satisfaction and potentially reduce the risk of PTSD. Birth satisfaction is a complex concept with many factors predicting it, such as time taken to hold baby, perceived threat to self, perceived threat to baby, birth type, perceived control [56] and the way women were treated by staff [68, 69]. Furthermore, predictors such as communication and information provision during birth are related to PTSD [70]. Therefore, healthcare professionals can improve birth satisfaction and potentially reduce the risk of PTSD through a range of actions and sensitive, clear communication, involving women in decision making and listening to their needs [71–75].

### Implications for research

The lack of prospective research carried out in this area makes conclusions difficult to draw. Measuring birth expectations during pregnancy, rather than after birth is more methodologically rigorous than retrospective measurement as it avoids recall bias. Furthermore, studies were variable in the methods used to measure a mismatch which also makes conclusions difficult to draw. Future research should focus on developing a standardised way of measuring this, to make cross-study comparisons easier. No prospective studies were identified that looked at the impact of a mismatch between birth expectations and experiences on birth partners, or women’s children. Additionally, research on physical outcomes was minimal. Therefore, more prospective research is needed in these areas to examine the gaps in this knowledge.

### Limitations

There are several limitations of this review that should be taken into consideration. For example, there are very few studies that evaluate the impact of a mismatch between birth expectations and experiences which makes conclusions difficult to draw. Most of the studies included evaluated the impact of a mismatch on birth satisfaction and psychological outcomes. Whilst the evidence is clearest for birth satisfaction and postnatal PTSD, the mixed results of the impact of a mismatch between birth expectations and experiences on other psychological outcomes suggests more prospective research is needed. Arguably, a limitation of the review methodology is the exclusion of studies where birth experiences and expectations were measured at the same time, or where a mismatch was not calculated. This may have meant some studies were missed. However, those studies that were identified and excluded supported the review findings [59–64]. Further, this decision meant that studies that were included avoided issues of recall bias and allowed a clear relationship between a birth experiences/expectation mismatch and the outcome variable to be ascertained. This approach may have also contributed to the high-quality methodology of the majority of the studies included in the review.

## Conclusion

Overall, this systematic review aimed to synthesise more methodologically rigorous evidence to understand whether unmet birth expectations are associated with adverse outcomes for women, their partners and their infants. The review found that the relationship between unmet birth expectations and depression/FOC and physical outcomes is not clear and more research is needed. However, the results from this review did identify that a mismatch between birth expectations and experiences is associated with birth satisfaction and may increase the risk of developing postnatal PTSD. Further prospective research is needed to identify examine gaps in knowledge and provide standardised methods of measuring childbirth expectations-experiences mismatch. To ensure women’s expectations are met, and therefore experience a satisfying birth experience, maternity providers should provide sensitive care, which acknowledges women’s needs and preferences, is based on open and clear communication, is delivered as early in pregnancy as possible, and enables women to make their own decisions about their care.

## Supplementary Information

**Additional file 1.**

## Data Availability

Data sharing is not applicable to this article as no datasets were generated or analysed during the current study.
