# Supplementary material for: When birth is not as expected: a systematic review of the impact of a mismatch between expectations and experiences
Source: BMC Pregnancy Childbirth. 2021 Jul 2;21:475. doi: 10.1186/s12884-021-03898-z (PMC8252193; doi:10.1186/s12884-021-03898-z)
Supplement: Supplementary file 1 — Additional file 1. [file 12884_2021_3898_MOESM1_ESM.docx]

**The impact of a mismatch between expectations and experiences of birth on postnatal outcomes: A systematic review**

**Supporting Information**

**Contents**

[1. Search syntax 2](#_Toc54175802)

[2. Quality appraisal of included studies 4](#_Toc54175803)

# **Search syntax and information sources**

| **Medline (n = 548)** |
| --- |
| TI ( health OR wellbeing OR problem* OR mental OR emotion* OR psychiatr* OR anxi* OR depress* OR affect* OR trauma OR PTSD OR stress OR ASD OR Disorder* OR illness OR symptom* OR breast* OR relationship OR bonding OR attachment OR child* OR infant ) AND TI ( pregnancy OR pregnant OR pre-nat* OR prepart* OR ante-nat* OR antenat* OR ante-part* OR peri-nat* OR perinat* OR peri-part* OR peripart* OR puerper* OR post-nat* OR postnat* OR post-part* OR postpart* OR mother* OR father* OR paternal OR maternal OR parent? OR spous* OR birth OR childbirth ) AND TI ( expect* OR belief OR desire OR predict* OR prefer* ) AND TI ( experienc* OR perception* OR perceiv* OR mismatch OR discrepancy OR satisfaction OR outcome OR mode OR "Obstetric outcome" ) |
| **Academic Search Complete (n = 606)** |
| TI ( health OR wellbeing OR problem* OR mental OR emotion* OR psychiatr* OR anxi* OR depress* OR affect* OR trauma OR PTSD OR stress OR ASD OR Disorder* OR illness OR symptom* OR breast* OR relationship OR bonding OR attachment OR child* OR infant ) AND TI ( pregnancy OR pregnant OR pre-nat* OR prepart* OR ante-nat* OR antenat* OR ante-part* OR peri-nat* OR perinat* OR peri-part* OR peripart* OR puerper* OR post-nat* OR postnat* OR post-part* OR postpart* OR mother* OR father* OR paternal OR maternal OR parent? OR spous* OR birth OR childbirth ) AND TI ( expect* OR belief OR desire OR predict* OR prefer* ) AND TI ( experienc* OR perception* OR perceiv* OR mismatch OR discrepancy OR satisfaction OR outcome OR mode OR "Obstetric outcome" ) |
| **CINAHL (n = 270)** |
| TI ( health OR wellbeing OR problem* OR mental OR emotion* OR psychiatr* OR anxi* OR depress* OR affect* OR trauma OR PTSD OR stress OR ASD OR Disorder* OR illness OR symptom* OR breast* OR relationship OR bonding OR attachment OR child* OR infant ) AND TI ( pregnancy OR pregnant OR pre-nat* OR prepart* OR ante-nat* OR antenat* OR ante-part* OR peri-nat* OR perinat* OR peri-part* OR peripart* OR puerper* OR post-nat* OR postnat* OR post-part* OR postpart* OR mother* OR father* OR paternal OR maternal OR parent? OR spous* OR birth OR childbirth ) AND TI ( expect* OR belief OR desire OR predict* OR prefer* ) AND TI ( experienc* OR perception* OR perceiv* OR mismatch OR discrepancy OR satisfaction OR outcome OR mode OR "Obstetric outcome" ) |
| **PsychArticles (n = 27)** |
| TI ( health OR wellbeing OR problem* OR mental OR emotion* OR psychiatr* OR anxi* OR depress* OR affect* OR trauma OR PTSD OR stress OR ASD OR Disorder* OR illness OR symptom* OR breast* OR relationship OR bonding OR attachment OR child* OR infant ) AND TI ( pregnancy OR pregnant OR pre-nat* OR prepart* OR ante-nat* OR antenat* OR ante-part* OR peri-nat* OR perinat* OR peri-part* OR peripart* OR puerper* OR post-nat* OR postnat* OR post-part* OR postpart* OR mother* OR father* OR paternal OR maternal OR parent? OR spous* OR birth OR childbirth ) AND TI ( expect* OR belief OR desire OR predict* OR prefer* ) AND TI ( experienc* OR perception* OR perceiv* OR mismatch OR discrepancy OR satisfaction OR outcome OR mode OR "Obstetric outcome" ) |
| **PsychInfo (n = 598)** |
| TI ( health OR wellbeing OR problem* OR mental OR emotion* OR psychiatr* OR anxi* OR depress* OR affect* OR trauma OR PTSD OR stress OR ASD OR Disorder* OR illness OR symptom* OR breast* OR relationship OR bonding OR attachment OR child* OR infant ) AND TI ( pregnancy OR pregnant OR pre-nat* OR prepart* OR ante-nat* OR antenat* OR ante-part* OR peri-nat* OR perinat* OR peri-part* OR peripart* OR puerper* OR post-nat* OR postnat* OR post-part* OR postpart* OR mother* OR father* OR paternal OR maternal OR parent? OR spous* OR birth OR childbirth ) AND TI ( expect* OR belief OR desire OR predict* OR prefer* ) AND TI ( experienc* OR perception* OR perceiv* OR mismatch OR discrepancy OR satisfaction OR outcome OR mode OR "Obstetric outcome" ) |
| **SCOPUS (n = 661)** |
| ( TITLE ( health  OR  wellbeing  OR  problem*  OR  mental  OR  emotion*  OR  psychiatr*  OR  anxi*  OR  depress*  OR  affect*  OR  trauma  OR  ptsd  OR  stress  OR  asd  OR  disorder*  OR  illness  OR  symptom*  OR  breast*  OR  relationship  OR  bonding  OR  attachment  OR  child*  OR  infant )  AND  TITLE ( pregnancy  OR  pregnant  OR  pre-nat*  OR  prepart*  OR  ante-nat*  OR  antenat*  OR  ante-part*  OR  peri-nat*  OR  perinat*  OR  peri-part*  OR  peripart*  OR  puerper*  OR  post-nat*  OR  postnat*  OR  post-part*  OR  postpart*  OR  mother*  OR  father*  OR  paternal  OR  maternal )  OR  TITLE ( parent?  OR  spous*  OR  birth  OR  childbirth )  AND  TITLE ( expect*  OR  belief  OR  desire  OR  predict*  OR  prefer* )  AND  TITLE ( experienc*  OR  perception*  OR  perceiv*  OR  mismatch  OR  discrepancy  OR  satisfaction  OR  outcome  OR  mode  OR  "Obstetric outcome" ) ) |
| **PubMed (n = 273)** |
| ((((health[Title] OR wellbeing[Title] OR problem*[Title] OR mental[Title] OR emotion*[Title] OR psychiatr*[Title] OR anxi*[Title] OR depress*[Title] OR affect*[Title] OR trauma[Title] OR PTSD[Title] OR stress[Title] OR ASD[Title] OR Disorder*[Title] OR illness[Title] OR symptom*[Title] OR breast*[Title] OR relationship[Title] OR bonding[Title] OR attachment[Title] OR child*[Title] OR infant[Title])) AND (pregnancy[Title] OR pregnant[Title] OR pre-nat*[Title] OR prepart*[Title] OR ante-nat*[Title] OR antenat*[Title] OR ante-part*[Title] OR peri-nat*[Title] OR perinat*[Title] OR peri-part*[Title] OR peripart*[Title] OR puerper*[Title] OR post-nat*[Title] OR postnat*[Title] OR post-part*[Title] OR postpart*[Title] OR mother*[Title] OR father*[Title] OR paternal[Title] OR maternal[Title] OR parent?[Title] OR spous*[Title] OR birth[Title] OR childbirth[Title])) AND (expect*[Title] OR belief[Title] OR desire[Title] OR predict*[Title] OR prefer*[Title])) AND (experienc*[Title] OR perception*[Title] OR perceiv*[Title] OR mismatch[Title] OR discrepancy[Title] OR satisfaction[Title] OR outcome[Title] OR mode[Title] OR "Obstetric outcome"[Title]) |
| **Web of Science* (n = 701)** |
| TITLE: (health OR wellbeing OR problem* OR mental OR emotion* OR psychiatr* OR anxi* OR depress* OR affect* OR trauma OR PTSD OR stress OR ASD OR Disorder* OR illness OR symptom* OR breast* OR relationship OR bonding OR attachment OR child* OR infant) *AND* TITLE: (pregnancy OR pregnant OR pre-nat* OR prepart* OR ante-nat* OR antenat* OR ante-part* OR peri-nat* OR perinat* OR peri-part* OR peripart* OR puerper* OR post-nat* OR postnat* OR post-part* OR postpart* OR mother* OR father* OR paternal OR maternal OR parent? OR spous* OR birth OR childbirth) *AND* TITLE: (expect* OR belief OR desire OR predict* OR prefer*) *AND* TITLE: (experienc* OR perception* OR perceiv* OR mismatch OR discrepancy OR satisfaction OR outcome OR mode OR "Obstetric outcome") |

* Science Citation Index Expanded (SCI-EXPANDED) – 1970-present; Social Sciences Citation Index (SSCI) – 1970-present; Art & Humanities Citation Index (A&HCI) – 1975-present; Conference Proceedings Citation Index-Science (CPCI-S) – 1990-present; Conference Proceedings Citation Index- Social Science & Humanities (CPCI-SSH) --1990-present; Book Citation Index– Science (BKCI-S) --2005-present; Book Citation Index– Social Sciences & Humanities (BKCI-SSH) --2005-present; Emerging Sources Citation Index (ESCI) --2015-present

# **Quality appraisal of included studies**

|  | **Q1** | **Q2** | **Q3** | **Q4** | **Q5** | **Q6** | **Q7** | **Q8** | **Q9** | **Score** | **Rating** |
| --- | --- | --- | --- | --- | --- | --- | --- | --- | --- | --- | --- |
| Fobelets et al. (2019) | Yes | Yes | Yes | Unclear | Yes | Yes | Yes | Unclear | Yes | 7/9 | High |
| Garthus-Niegel et al. (2014) | Yes | Yes | Yes | Yes | Yes | Yes | Yes | Unclear | Yes | 8/9 | High |
| Houston et al. (2015) | Yes | Yes | Yes | Yes | Yes | Yes | Unclear | Unclear | Yes | 7/9 | High |
| Mei et al. (2016) | Yes | No | N/A | Yes | Unclear | Unclear | Unclear | No | Yes | 3/8 | Low |
| Preis et al. (2019) | Yes | Yes | Yes | Yes | Yes | Yes | Yes | No | Yes | 8/9 | High |
| Sluijs et al. (2020) | Yes | Yes | Yes | Yes | Yes | Yes | Yes | Yes | Yes | 9/9 | High |
| Stein DeLuca & Lobel (2014) | Yes | Yes | Yes | Yes | Yes | Yes | Yes | No | Yes | 8/9 | High |
| Tanglakmankhong (2010) | Yes | Yes | Yes | Yes | Yes | Unclear | Yes | Yes | Yes | 8/9 | High |
| Philipson-Price (1982) | Yes | Yes | Unclear | Yes | Unclear | Yes | Yes | N/A | Yes | 6/8 | Medium |

Note. Q1. Was the exposure measured in a valid and reliable way? Q2. Were confounding factors identified? Q3. Were strategies to deal with confounding factors stated? Q4. Were the participants free of the outcome at the start of the study? Q5. Were the outcomes measured in a valid and reliable way? Q6. Was the follow up time reported and sufficient to be long enough for outcomes to occur? Q7. Was follow up complete, if not, were the reasons to loss to follow up described? Q8. Were strategies to address incomplete follow up utilised? Q9. Was appropriate statistical analysis used?
